# Homocysteine and Folic Acid: Risk Factors for Alzheimer's Disease—An Updated Meta-Analysis

**DOI:** 10.3389/fnagi.2021.665114

**Published:** 2021-05-26

**Authors:** Qianwen Wang, Jingjing Zhao, Hongtao Chang, Xu Liu, Ruixia Zhu

**Affiliations:** Department of Neurology, The First Affiliated Hospital of China Medical University, Shenyang, China

**Keywords:** Alzheimer's disease, vascular dementia, folic acid, homocysteine, meta-analysis

## Abstract

**Background:** Recent studies have reported that homocysteine (Hcy) may play a vital role in the pathogenesis of vascular dementia (VaD) and Alzheimer's disease (AD). Our study explored the relationship between the plasma Hcy and folate levels and the risk of dementia.

**Methods:** We searched Embase, PubMed, and Web of Science for published literature, including case-control studies and prospective cohort studies, and performed a systematic analysis.

**Results:** The results of our meta-analysis, consisting of case-control studies, showed higher levels of Hcy and lower levels of folate in dementia, AD, and VaD patients than those in non-demented controls (for dementia: SMD = 0.812, 95% CI [0.689, 0.936], *p* = 0.000 for Hcy; SMD = −0.677, 95% CI [−0.828, −0.525], *p* = 0.000 for folate). AD patients showed significantly lower plasma Hcy levels compared to VaD patients (SMD = −0.278, 95% CI [−0.466, −0.09], *p* = 0.000). Subgroup analysis revealed that ethnicity, average age, and dementia type had no significant effect on this association. Furthermore, from the analysis of prospective cohort studies, we identified that elevated plasma Hcy levels were associated with an increased risk of dementia, AD, and VaD (RR_dementia_ = 1.22, 95% CI [1.08, 1.36]; RR_AD_ = 1.07, 95% CI [1.04, 1.11]; RR_VaD_ = 1.13, 95% CI [1.04, 1.23]). In addition, every 5 μmol/L increase in the plasma Hcy level was associated with a 9% increased risk of dementia and a 12% increased risk of AD.

**Conclusion:** Hcy and folic acid are potential predictors of the occurrence and development of AD. A better understanding of their function in dementia could provide evidence for clinicians to rationalize clinical intervention strategies.

## Introduction

Dementia is a severe threat to the health of older people and places a heavy burden on their families and society (Brookmeyer et al., 2007). Alzheimer's disease (AD) is the most common form of dementia and is characterized by β-amyloid (Aβ)-containing plaques and neurofibrillary tangles (NFT) (Lane et al., 2018). Vascular dementia (VaD) is the second most common form of dementia and has similar clinical manifestations that are caused by specific ischemic lesions, such as thalamus and white matter. There has not yet been an effective therapy for dementia, and it is urgent to identify considerable methods to delay cognitive decline. It has been reported that subjects with vascular risk factors are more susceptible to VaD and AD. Plasma total homocysteine (Hcy), as a common risk factor for vascular diseases, could be modified by supplementation with folic acid and B vitamin as well as relevant with dementia (Beydoun et al., 2014).

Several recent studies have focused on the role of Hcy, vitamin B12, and folate as possible participants in the neurodegenerative processes. Hcy is a sulfur-containing amino acid produced during methionine metabolism. Hcy remethylation requires folate as the methyl donor and vitamin B12 as the co-factor; deficiencies of vitamin B12 or folate would lead to hyperhomocysteinemia. Additionally, Hcy can also be converted to cystathionine via transsulfuration (Joosten, 2001). Thus, Hcy and folate play an important role in the transfer of methyl groups in cellular metabolism, which contributes to the pathogenesis of AD (Smith and Refsum, 2016).

The association between Hcy levels and dementia risk has been studied for many years. However, the results remain controversial (Clarke et al., 1998; Leblhuber et al., 2000; Miller et al., 2002; Quadri et al., 2004). A previous meta-analysis by Van Dam et al. demonstrated higher Hcy levels and lower vitamin B12 and folic acid levels in AD patients (Van Dam and Van Gool, 2009). They also indicated that folate, vitamin B6, and vitamin B12 levels were related to the risk of developing AD. Subsequently, a meta-analysis of relevant epidemiological studies by Beydoun et al. indicated that a high Hcy level represented a modifiable risk factor for cognitive dysfunction and was a strong predictor of the incidence of AD (Beydoun et al., 2014). A study of 1,249 elderly patients performed by Ariogul et al. failed to find any correlation between Hcy levels, folate levels, and cognitive function (Ariogul et al., 2005). Another study by Faux et al. showed that Hcy levels were higher in female AD patients compared to those in female healthy control subjects, however, that was not observed in male subjects (Faux et al., 2011). Moreover, current researches also found that factors of ethnicity and age might contribute to the inheritance and development of dementia. Therefore, we performed an updated comprehensive meta-analysis to further evaluate the association of plasma Hcy and folate levels with dementia risk covering all currently available data in order to provide evidence for clinical practice. To achieve this, we first performed a systematic analysis of case–control studies on Hcy and folate levels in dementia patients and divided these into subgroups according to dementia type, geographical ethnicity, average age, and gender distribution. We then focused on prospective studies to explore the association between Hcy levels and the risk of developing AD and VaD.

## Materials and Methods

### Search Strategy

We searched the electronic databases PubMed, Web of Science, and EMBASE to identify suitable studies published prior to December 2020 using specific keywords, using the following search strategies: (1) Homocysteine OR “Hcy” OR Hyperhomocysteinemia OR Folate OR Folic acid and (2) Dementia OR Cognitive OR Cognition OR Alzheimer OR Vascular dementia. Some articles were identified via manual screening of relevant references from other studies on the subject.

#### Inclusion and Exclusion Criteria

The inclusion criteria for the meta-analysis of case-control studies were that the studies must provide the number of dementia patients and controls and clearly present means and standard deviations (SD) of Hcy and/or folic acid levels for both patients and controls. For the meta-analysis of prospective cohort studies, studies must present blood Hcy concentration levels, adjusted relative risk (RR), and 95% confidence intervals (CI) for dementia or AD occurrence.

The exclusion criteria were as follows: (1) we selected population-based case-control studies and cohort studies to extract raw data, case report, review, letters were all excluded; (2) we designated two independent reviewers to scrutinize the manuscripts of retrieval, irrelevant titles and abstracts were excluded; and (3) studies with incomplete data were excluded such as undefined included cohorts and imprecise level of Hcy and folic acid.

### Study Quality and Data Extraction

Two reviewers independently assessed the included studies. The extracted data included the name of the first author, publication year, the ethnicity of samples, the number of subjects, gender distribution, mean age, mean follow-up duration, mean and SD of Hcy and folate levels for patients and controls, source and detection method used for Hcy and folate analyses, categories of plasma Hcy, and multivariable-adjusted effects (RR and 95% CI for each exposure category). Different Hcy and folate concentration measurement units were converted to a standard format (μmol/L for Hcy and nmol/L for folate).

The Newcastle-Ottawa Scale (NOS) (Stang, 2010) was used to assess the quality of the included studies. The NOS contains three domains (study selection, group comparability, and cohort exposure) covering four, two, and three points. The total NOS score ranged from zero to nine stars, and six to nine points were considered high quality. All the selected articles scored six or more stars in this system (Supplementary Table 1).

All the included studies were approved by ethics committees and conducted according to the Code of Ethics of the World Medical Association (Declaration of Helsinki). All the participants of the included studies provided written informed consent.

### Statistical Analysis

The standard mean differences (SMD) with a 95% confidence interval (CI) were calculated to explore the differences in Hcy and folate levels between the dementia and control groups. The pooled RR with 95% CI was used to evaluate the relationship between the Hcy and folate levels and the risk of dementia. Heterogeneity was evaluated using the *Q*-test and the *I*^2^-test. *I*^2^ > 50% represented high heterogeneity. Thus, the random-effects model was applicable. Subgroup analyses—stratified by dementia type (AD and VaD), geographical ethnicity (Caucasian or Asian), average age (60 ≤ age < 70, 70 ≤ age < 80, or age ≥ 80), and gender distribution (male/total ≥ 50%, male/total < 50%)—were also conducted. A dose-response meta-analysis was used to assess the potential linear and non-linear dose-response relationships between blood Hcy levels and the risk of dementia. Sensitivity analysis was used to assess the results after removing any of the studies one at a time. The risk of publication bias was assessed using Egger's test and Begg's test. A value of *p* ≥ 0.05 indicated no publication bias. If publication bias was observed, we applied the trim and fill method to recalculate the results. All meta-analyses were performed using Stata version 15.1.

## Results

### Study Selection and Characteristics

**Figure 1:**
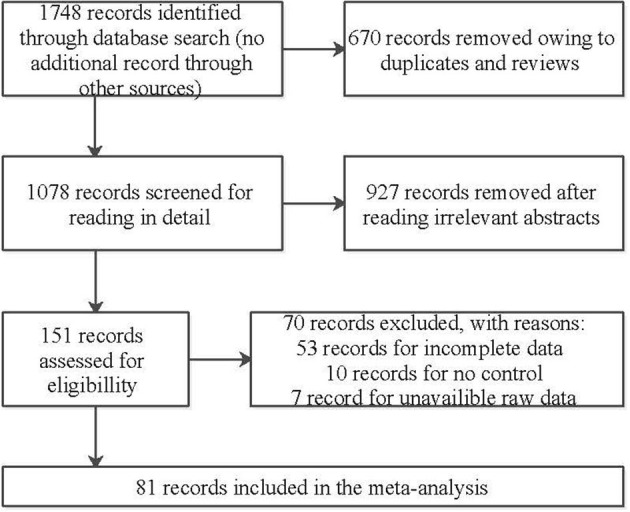
Flowchart of study selection in our meta-analysis.

We initially identified 1,746 relevant articles from available databases, including Embase, Pubmed, and Web of Science, and 1,078 of the articles were left after removing duplications and reviews. We excluded 927 articles after reading titles or abstracts and selected 151 full-text articles for eligibility. Subsequently, 70 articles were removed on account of failing to data extraction after careful perusal, thus 81 articles were included in this meta-analysis eventually. Thereinto, 55 and 37 articles were retrospective researches relevant to Hcy and folic acid, and 13 articles and 5 articles were prospective aiming at Hcy and folic acid, respectively. A flow diagram of the retrieval process is illustrated in Figure 1. The baseline characteristics of the included studies are presented in Tables 1, 2, Supplementary Tables 3, 4. In our study, serum or plasma folate and plasma Hcy levels were studied. A total of 3,240 dementia patients (2,932 for AD and 308 for VaD) and 4,901 non-dementia controls were enrolled for the analysis of folate. Five thousand one hundred and fifty-one cases (4,547 for AD and 604 for VaD) and 5,113 controls were examined for Hcy. In addition, 15,134 samples and 1,771 cases were enrolled in our meta-analysis, based on information obtained from prospective studies (Supplementary Table 2).

**Table 1:** The baseline characteristics of included studies (Hcy).

**Reference**	**Country**	**Method**	**AD**			**Controls**		
			***N* (male)**	**Age**	**Hcy (umol/L)**	***N* (male)**	**Age**	**Hcy**
Clarke et al. (1998)	Caucasian	HPLC	164 (64)	73.2 ± 8.6	15.3 ± 8.4	108 (46)	72.8 ± 8.8	13.2 ± 4
Fekkes et al. (1998)	Caucasian	HPLC	14 (4)	73.6 ± 6.3	19.4 ± 9.2	17 (17)	70.1 ± 1.3	17.9 ± 3.5
Leblhuber et al. (2000)	Caucasian	FPIA	19 (4)	74.8 ± 8.8	17.8 ± 6.6	19 (8)	70.2 ± 8.8	13.8 ± 4.2
Pollak et al. (2000)	Caucasian	amino acid analysis	92 (20)	85 ± 7.2	11.9 ± 5.6	82 (21)	82 ± 8.2	14 ± 7.1
Bottiglieri et al. (2001)	Caucasian	HPLC	48 (16)	71 ± 8.5	12.4 ± 11.1	14 (9)	40.6 ± 14.6	7.1 ± 2.9
Postiglione et al. (2001)	Caucasian	HPLC	74 (45)	68 ± 8	20.9 ± 15.0	74 (42)	68 ± 7	11.8 ± 5.0
Nilsson et al. (2002)	Caucasian	HPLC	28	60.8 ± 3.8	12.7 ± 3.2	26	61.3 ± 5.5	12.3 ± 2.8
Nilsson et al. (2002)	Caucasian	HPLC	94	77.6 ± 6.4	18.5 ± 9.3	36	79.9 ± 3.7	15.5 ± 3.7
Hogervorst et al. (2002)	Caucasian	HPLC	137 (55)	73.9 ± 9	14.7 ± 4.9	277 (139)	73.3 ± 7.7	12.8 ± 3.9
Miller et al. (2002)	Caucasian	HPLC	32 (13)	78 ± 7	10.6 ± 2.0	22 (8)	75 ± 7	9.3 ± 2.2
Selley et al. (2002)	Caucasian	Capillary column gas chromatography	27 (14)	77.4	21.05 ± 3.74	25 (12)	78.4	16.01 ± 3.9
Selley (2003)	Caucasian	Capillary column gas chromatography	25 (13)	76	23.52 ± 4.6	25 (12)	74.6	19.04 ± 3.55
Mizrahi et al. (2003)	Caucasian	automated IA	64	/	12.3 ± 4.3	64	/	11.5 ± 3.7
Nagga et al. (2003)	Caucasian	FPIA	47 (20)	74.7 ± 7.3	16.5 ± 6.4	101 (52)	69.0 ± 5.8	12.9 ± 4.2
Religa et al. (2003)	Caucasian	FPIA	99	74.2 ± 6.3	18.03 ± 9.94	100	71.2 ± 6	14.43 ± 4.48
Gallucci et al. (2004)	Caucasian	HPLC	137 (46)	76.9 ± 6.8	21.4 ± 10.6	42 (16)	76.8 ± 9.7	15.5 ± 5.2
Genedani et al. (2004)	Caucasian	/	22	81.9 ± 1.7	22.5 ± 2.0	22	81.1 ± 1.3	16.3 ± 1.15
Mizrahi et al. (2004)	Asian	FPIA	75 (29)	88 ± 7.0	20.6 ± 8.7	155 (75)	76 ± 7.0	16.4 ± 6.5
Quadri et al. (2004)	Caucasian	FPIA	74 (25)	79.1 ± 7.7	16.8 ± 7.0	55 (21)	75.6 ± 8.5	14.6 ± 6.1
Anello et al. (2004)	Caucasian	FPIA	180	71.0 ± 6.6	13.9 ± 9.2	181	69.5 ± 12.7	11.0 ± 5.3
Malaguarnera et al. (2004)(1)	Caucasian	HPLC	22 (7)	72.6 ± 7.38	22.3 ± 4.51	24 (12)	73.7 ± 4.20	10.7 ± 3.00
Malaguarnera et al. (2004)(2)	Caucasian	HPLC	30 (10)	71.3 ± 7.99	23.5 ± 4.62	30 (13)	73.6 ± 4.14	10.8 ± 2.92
Irizarry et al. (2005)	Caucasian	HPLC	145 (65)	75.9 ± 8.7	8.9 ± 3.2	88 (38)	70.3 ± 9.8	8.7 ± 3.2
Dominguez et al. (2005)	Caucasian	FPIA	29 (6)	73.35 ± 5.36	18.35 ± 4.2	19 (12)	73.89 ± 8.87	11.11 ± 1.88
Folin et al. (2005)	Caucasian	HPLC	79	80.33 ± 7.06	21.01 ± 7.80	24	71.24 ± 9.69	15.79 ± 5.55
Zhang et al. (2005)	Asian	High voltage capillary electrophoresis	105 (55)	71.2 ± 6.7	16.04 ± 3.84	102 (52)	69.5 ± 7.0	11.94 ± 3.87
Guidi et al. (2005)	Caucasian	FPIA	97	73	19.0 ± 7.88	23	71	13.0 ± 3.36
Asita De Silva et al. (2005)	Asian	FPIA	23 (8)	72 ± 6.8	13.3 ± 5.3	21 (8)	70.5 ± 3.9	8.3 ± 2.2
Quadri et al. (2005)	Caucasian	FPIA	111	78.9 ± 7.5	16.9 ± 7.3	79	75.0 ± 8.5	14.4 ± 6.1
Guidi et al. (2006)	Caucasian	FPIA	71 (23)	78 (55–92)	19.10 ± 7.16	44 (10)	73 (54–93)	12.89 ± 4.11
da Silva et al. (2006)	Caucasian	HPLC	42 (7)	73.8 ± 7.2	18.31 ± 7.6	50 (13)	73.9 ± 6.5	15.34 ± 5.4
Hernanz et al. (2007)	Caucasian	HPLC	25 (10)	73.2 ± 7.1	14.8 ± 7.4	44	73.5 ± 3.2	10.4 ± 2.7
Selley (2007)	Caucasian	LC–MS/MS	29 (15)	71.9	15.62 ± 3.66	26 (14)	71.3	9.57 ± 4.28
Koseoglu and Karaman (2007)	Caucasian	HPLC	51 (21)	78.25 ± 4.14	14.2 ± 2.97	40 (17)	76.13 ± 3.88	10.3 ± 1.28
Davis et al. (2007)	Caucasian	FPIA	19	/	10.35 ± 2.27	46	69.5 ± 6.1719	9.85 ± 2.17
Kim et al. (2008a)	Asian	HPLC	86 (15)	77.6 ± 6.8	14.2 ± 7.2	625 (273)	72.0 ± 5.3	12.5 ± 5.4
Galimberti et al. (2008)	Caucasian	isocratic rp-HPLC	29	78.45 ± 4.63	14.60 ± 5.90	23	70.13 ± 3.01	11.53 ± 3.90
Hagnelius et al. (2008)	Caucasian	FPIA	42	72.7 ± 10.1	13.8 ± 4.6	73	64.1 ± 9.5	11.9 ± 3.6
Villa et al. (2009)	Caucasian	HPLC/fluorescence detection	16 (7)	70.8 ± 7.79	13.9 ± 2.47	15 (7)	74.7 ± 6.73	9.1 ± 2.82
Villa et al. (2009)	Asian	EIA	51 (33)	75 ± 16	20.4 ± 16.5	49 (26)	68 ± 8	14.5 ± 5
Linnebank et al. (2010)	Caucasian	Particle-enhanced immunonephelometry	60 (17)	73 ± 8	14.1 ± 4.3	60 (36)	62 ± 10	12.7 ± 5.4
Tu et al. (2010)	Asian	FPIA	92 (30)	73.77 ± 9.4	12.9 ± 7.3	67 (27)	71.7 ± 5.8	12.1 ± 4.4
Smach et al. (2011)	Caucasian	FPIA	70 (39)	73.2 ± 6.9	12.9 ± 3.6	30 (19)	73.5 ± 6.8	11.6 ± 1.7
Ferlazzo et al. (2011)	Caucasian	HPLC	51	/	18.7 ± 4.0	69	75.6 ± 5.1	12.7 ± 3.1
Czapski et al. (2012)	Caucasian	/	182	/	16.04 ± 7.53	90	/	12.62 ± 3.94
Mansoori et al. (2012)	Asian	competitive IA	80 (54)	66.3 ± 9.2	15.39 ± 7.2	120 (75)	63.8 ± 8.2	13.55 ± 5.4
Yesil et al. (2012)	Caucasian	/	126 (44)	76.2 ± 6.8	17.5 ± 9.9	286 (123)	75.2 ± 6.3	17.9 ± 7.7
Kim et al. (2013)	Asian	HPLC	100 (26)	79.4 ± 6.8	11.9 ± 3.9	121 (54)	71.4 ± 6.6	9.2 ± 3.7
Elhawary et al. (2013)	Caucasian	FPIA	43 (14)	69.2 ± 8.1	18.4 ± 6.3	32 (10)	70.7 ± 8.8	13.0 ± 3.8
Cervellati et al. (2013)	Caucasian	ROCHE COBAS INTEGRA	89 (23)	78.8 ± 0.8	16.8 ± 1.7	48 (15)	77.8 ± 0.7	14.6 ± 3.4
Choe et al. (2014)	Asian	CEIA	24 (3)	69.25 ± 8.56	9.83 ± 3.64	14 (8)	72.64 ± 5.49	7.79 ± 1.53
Madsen et al. (2015)	Caucasian	Validated enzyme immunoassay	186 (97)	75.45 ± 6.84	10.77 ± 3.32	225 (117)	75.33 ± 7.67	9.94 ± 2.80
Doody et al. (2015)	Caucasian	Recombinant cycling assay	197 (68)	77.41 ± 8.29	16.21 ± 9.01	198 (63)	70.42 ± 8.89	13.3 ± 5.03
Ma et al. (2017)	Asian	Enzymatic conversion method	89 (33)	74.62 ± 8.01	16.37 ± 7.46	115 (46)	72.82 ± 8.87	13.21 ± 4.05
Lanyau-Domínguez et al. (2020)	Caucasian	Enzymatic oxidation	40	82.8	18.0 ± 7.7	214	78	13.6 ± 6.5
Sutovsky et al. (2020)	Caucasian	competitive IA	564	72.4 ± 7.0	15.7 ± 4.2	534	71.5 ± 6.7	13.7 ± 4.5

*FPIA, fluorescence polarization immunoassay; EIA, enzyme immunoassay; CEIA, chemiluminescent microparticle immunoassay*.

**Table 2:** The baseline characteristics of included studies (folic acid).

**Reference**	**Ethnicity**	**Source**	**Method**	**AD**			**Controls**		
				***N* (male)**	**Age**	**Folic acid (nM/L)**	***N* (male)**	**Age**	**Folic acid (nM/L)**
Parnetti et al. (1992)	Caucasian	Plasma	RIA	52	75.08 ± 1.2	9.47 ± 5.75	26	72.1 ± 1.4	14.07 ± 5.79
Joosten (2001)	Caucasian	Serum	RIA	52	82.8 ± 4.9	7.9 ± 4.2	49	79 ± 5.9	8.6 ± 3.2
Clarke et al. (1998)	Caucasian	Serum	Microbiological assay	164 (64)	73.2 ± 8.6	17.6 ± 10.7	108 (46)	72.8 ± 8.8	22.9 ± 10
Leblhuber et al. (2000)	Caucasian	Serum	RIA	19 (4)	74.8 ± 8.8	9.988 ± 3.405	19 (8)	70.2 ± 8.8	14.301 ± 9.307
Bottiglieri et al. (2001)	Caucasian	Plasma	RIA	48 (16)	71 ± 8.5	8.0 ± 3.4	14 (9)	40.6 ± 14.6	12.1 ± 10.0
Selley et al. (2002)	Caucasian	Plasma	RIA	27 (14)	77.4	14.74 ± 4.26	25 (12)	78.4	25.09 ± 4.7
Religa et al. (2003)	Caucasian	Serum	Folate reagent assay	99	74.2 ± 6.3	19.32 ± 7.67	100	71.2 ± 6	17.16 ± 12.24
Mizrahi et al. (2004)	Asian	Plasma	RIDA	75 (29)	88 ± 7.0	4.3 ± 3.2	155 (75)	76 ± 7.0	4.8 ± 2.6
Quadri et al. (2004)	Caucasian	Serum	RIA	74 (25)	79.1 ± 7.7	13.6 ± 5.6	55 (21)	75.6 ± 8.5	16.9 ± 5.8
Anello et al. (2004)	Caucasian	Serum	MEIA	180	71.0 ± 6.6	14.3 ± 5.7	181	69.5 ± 12.7	15.7 ± 5.9
Malaguarnera et al. (2004)(1)	Caucasian	Serum	RA	22 (7)	72.6 ± 7.38	10.0 ± 2.72	24 (12)	73.7 ± 4.20	13.9 ± 3.03
Malaguarnera et al. (2004)(2)	Caucasian	Serum	RA	30 (10)	71.3 ± 8.0	10.6 ± 3.16	30 (13)	73.6 ± 4.1	13.6 ± 3.18
Ravaglia et al. (2004)	Caucasian	Serum	ECLIA	51 (16)	86.7 ± 5.4	11.1 ± 4.3	29 (13)	86.7 ± 5.9	16.57 ± 7.26
Gallucci et al. (2004)	Caucasian	Serum	Chemiluminescence	137 (46)	76.9 ± 6.8	11.58 ± 6.13	42 (12)	76.8 ± 9.7	14.07 ± 11.12
Dominguez et al. (2005)	Caucasian	Serum	Ionic capture assay	29 (6)	73.35 ± 5.36	17.91 ± 7.20	19 (12)	73.89 ± 8.87	29.62 ± 8.99
Quadri et al. (2005)	Caucasian	Serum	RIA	111	78.9 ± 7.5	13.1 ± 5.9	79	75.0 ± 8.5	16.8 ± 5.5
Lovati et al. (2007)	Caucasian	Serum	CLIA	108 (27)	76.6 ± 7.5	8.22 ± 5.33	76 (18)	67.6 ± 7.2	15.59 ± 7.95
Kim et al. (2008a)	Asian	serum	IA	86 (15)	77.6 ± 6.8	23.2 ± 10.2	625 (273)	72.0 ± 5.3	24.7 ± 12.7
Koseoglu and Karaman (2007)	Caucasian	Serum	Competitive CLIA	51 (21)	78.25 ± 4.14	21.45 ± 4.40	40 (17)	76.13 ± 3.88	28.15 ± 3.41
Hagnelius et al. (2008)	Caucasian	Serum	Solid-phase time-resolved FIA	42	72.7 ± 10.1	11.2 ± 4.9	73	64.1 ± 9.5	13.4 ± 5.8
Galimberti et al. (2008)	Caucasian	Serum	Competitive CLIA	29	78.45 ± 4.63	8.65 ± 2.81	23	70.13± 3.01	19.86± 6.17
Villa et al. (2009)	Asian	Serum	RIA	51 (33)	75 ± 16	14.53 ± 6.58	49 (26)	68 ± 8	15.89 ± 8.63
Villa et al. (2009)	Caucasian	Plasma	Chemiluminescent	16 (7)	70.8 ± 7.79	16.80 ± 4.70	15 (7)	74.7 ± 6.73	19.07 ± 4.09
Bi et al. (2009)	Asian	Plasma	Microbiological assay	106 (55)	/	11.92 ± 5.36	104 (49)	/	15.28 ± 6.95
Linnebank et al. (2010)	Caucasian	Serum	Competitive CLIA	60 (17)	73 ± 8	15.62 ± 7.04	60 (36)	62 ± 10	14.05 ± 7.74
Morillas-Ruiz et al. (2010)	Caucasian	Serum	ECLIA	48 (13)	76.5 ± 3.5	21.8 ± 8.7	52 (12)	79 ± 4	28.8 ± 7.7
Hooshmand et al. (2010)	Caucasian	Serum	Chemiluminescent microparticle folate binding protein assay	17 (4)	73.4 ± 4.3	7.9 ± 3.4	254 (99)	70.5 ± 3.5	7.1 ± 3.9
Faux et al. (2011)	Caucasian	Serum	Chemiluminescent	205 (80)	78.4 ± 8.7	29.35 ± 14.46	760(323)	70 ± 7	30.29 ± 12.68
Czapski et al. (2012)	Caucasian	Serum	/	204	–	19.86 ± 17.93	99	–	19.48 ± 8.54
Yesil et al. (2012)	Caucasian	/	/	126 (44)	76.2 ± 6.8	24.29 ± 12.26	286 (123)	75.2 ± 6.3	25.20 ± 12.26
Piazza et al. (2012)	Caucasian	Serum	Competitive CLIA	107	73.9 ± 5.0	13.17 ± 5.45	100 (57)	71.9 ± 5.4	15.66 ± 4.99
Kim et al. (2013)	Asian	Serum	RIA	100	79.4 ± 6.8	12.94 ± 10.67	121	71.4 ± 6.6	13.85 ± 9.31
Mansoori et al. (2014)	Asian	Serum	Competitive IA	80 (54)	66.3 ± 8.9	16.57 ± 7.95	120 (75)	63.8 ± 8.2	19.98 ± 8.17
Ma et al. (2017)	Asian	Serum	Automated CLIA	89 (33)	74.62 ± 8.01	11.65 ± 8.10	115 (46)	72.82 ± 8.87	15.96 ± 8.35
Soni et al. (2018)	Asian	Serum	/	18 (10)	74.5 ± 8.54	22.04 ± 9.87	18 (10)	70.94 ± 7.4	21.79 ± 3.04
Meng et al. (2019)	Asian	Serum	RA	182 (50)	68.84 ± 7.63	8.65 ± 6.37	728 (424)	68.86 ± 7.69	9.35 ± 7.32
Lanyau-Domínguez et al. (2020)	Caucasian	Serum	ECLIA	37	82.8	20.88 ± 13.17	228	78	24.06 ± 11.58

*RIA, radioimmunoassay; RA, radioassay; IA, immunoassay; FIA, fluoroimmunoassay; MEIA, microparticle enzyme immunoassay; ECLIA, electrochemiluminescence immunoassay; CLIA, chemiluminescent immunoassay; RIDA, radioisotope dilution assay*.

### Meta-analysis

#### Plasma Hcy and Folate Levels in Dementia Patients

**Figure 2:**
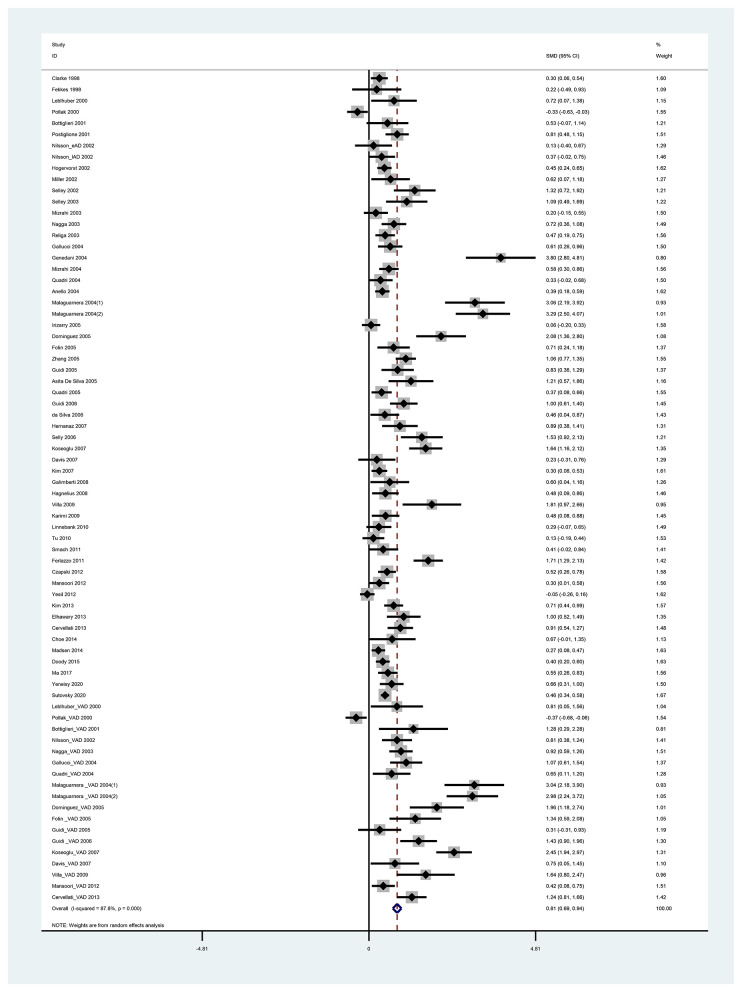
Forest plot of standard mean difference (SMD) and 95% confidence interval (95%CI) in dementia and control group for plasma homocysteine.

**Figure 3:**
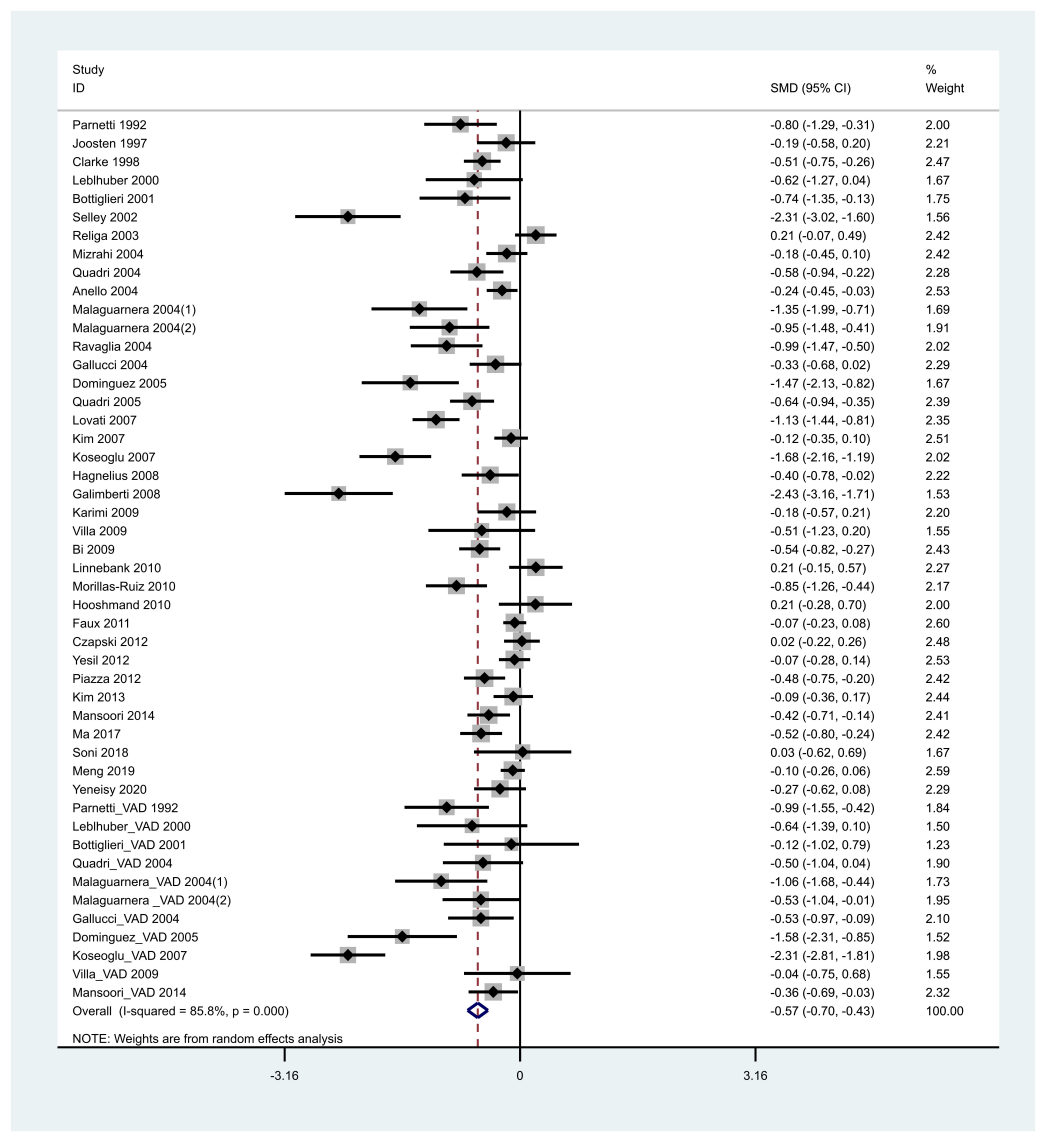
Forest plot of standard mean difference (SMD) and 95% confidence interval (95%CI) in dementia and control group for folic acid.

We discovered that heterogeneity in the SMD of Hcy and folate existed in all sets of the analyzed groups among various studies (*P* < 0.05) (Supplementary Figure 1). Further, a random-effects model was used to take additional account of study variation. Our study found that plasma Hcy levels were higher in dementia patients than in controls, with an SMD of 0.812 (95% CI [0.689–0.936], *p* = 0.000) (Figure 2). Our study also found that folate levels were lower in dementia patients than in controls, with an SMD of −0.568 (95% CI [−0.705, −0.431], *p* = 0.000) (Figure 3). Begg's and Egger's tests indicated publication bias (*p* = 0.000 and *p* = 0.000, respectively). Therefore, we used the trim and fill method to recalculate the pooled effect size, and the results were slightly altered but still similar to our original risk estimate (SMD = 0.812, 95% CI [0.689, 0.936], *p* = 0.000 for Hcy; SMD = −0.677, 95% CI [−0.828, −0.525], *p* = 0.000 for folate) (Supplementary Figure 2). Sensitivity analysis revealed that the results were identical to the primary results after removing any one of the studies one at a time. This indicated that no single study exerted a substantial influence on the pooled effect size (ES) (Supplementary Figures 3, 4).

#### Subgroup Analysis

To further explore the effect of geographical ethnicity, average age, gender, and dementia type on plasma Hcy and folate levels in dementia patients and controls, subgroup analyses were performed. The 55 studies for Hcy and 37 studies for folate levels were divided into two subgroups based on the dementia type. Subgroup analysis showed lower folic acid levels in AD patients, with an SMD of −0.503 (95% CI [−0.644, −0.362], *p* < 0.05), and higher Hcy levels in AD patients, with an SMD of 0.689 (95% CI [0.569, 0.809], *p* < 0.05), than in the controls. This indicated significant differences in plasma Hcy and folate levels between AD patients and controls. Further, we also found that Hcy levels were higher and folate levels were lower in VaD patients than in the controls. Additionally, high Hcy and low folic acid levels in dementia patients were found in the Caucasian and Asian cohorts. Ethnicity also exhibited no significant effect on this association. Further subgroup analysis based on age (age 60–70, SMD = 0.519 [0.288, 0.75], *p* = 0.000; age 70–80, SMD = 0.826 [0.695, 0.959], *p* = 0.000; age 80–90, SMD = 0.961 [0.438, 1.485], *p* = 0.000) showed higher levels of Hcy in dementia patients than in healthy controls. In both male and female subgroups, higher Hcy and lower folic acid levels were observed in dementia patients than in controls. Plasma folate levels were significantly lower in dementia patients with a mean age of 70–80 and 80–90 years than in controls. However, dementia patients with an average age of 60–70 years had marginally lower folic acid levels (SMD = −0.262, [−0.486, −0.037], *p* = 0.022). The above results are shown in Supplementary Figures 5–12, Table 3.

**Table 3:** Summary of statistic results in our meta-analysis and subgroup analysis.

	**Subgroup**	**SMD**	**95% CI**	***P*-value**	**Heterogeneity**
Hcy	AD	0.689	[0.569, 0.809]	0.000	85.2%
	VAD	1.228	[0.818, 1.639]	0.000	90.9%
	Caucasian	0.872	[0.727, 1.016]	0.000	89%
	Asian	0.546	[0.368, 0.723]	0.000	69.1%
	Age 60–70	0.519	[0.288, 0.75]	0.000	50.4%
	Age 70–80	0.826	[0.695, 0.959]	0.000	86.7%
	Age 80–90	0.961	[0.438, 1.485]	0.000	94.2%
	AD vs. VAD Male Female	−0.278 1.24 0.71	[−0.466, −0.09] [0.91–1.58] [0.53–0.88]	0.004 0.000 0.000	65.7% 90.7% 87.9%
Folate	AD	−0.503	[−0.644, −0.362]	0.000	85.5%
	VAD	−0.796	[−1.2, −0.393]	0.000	82.6%
	Caucasian	−0.676	[−0.856, −0.497]	0.000	87.8%
	Asian	−0.256	[−0.377, −0.314]	0.000	46.2%
	Age 60–70	−0.262	[−0.486, −0.037]	0.022	85.8%
	Age 70–80	−0.574	[−0.731, −0.418]	0.000	85.4%
	Age 80–90	−0.69	[−1.190, −0.193]	0.000	90.5%
	AD vs. VAD Male Female	0.032 −0.64 −0.57	[−0.132, 0.196] [−0.97, −0.3] [−0.74, −0.39]	0.703 0.000 0.000	20.2% 84.1% 85.4%

*SMD, standard mean differences; 95 %CI, confidence interval*.

#### Comparison of Hcy and Folic Acid Levels in AD and VaD Patients

**Figure 4:**
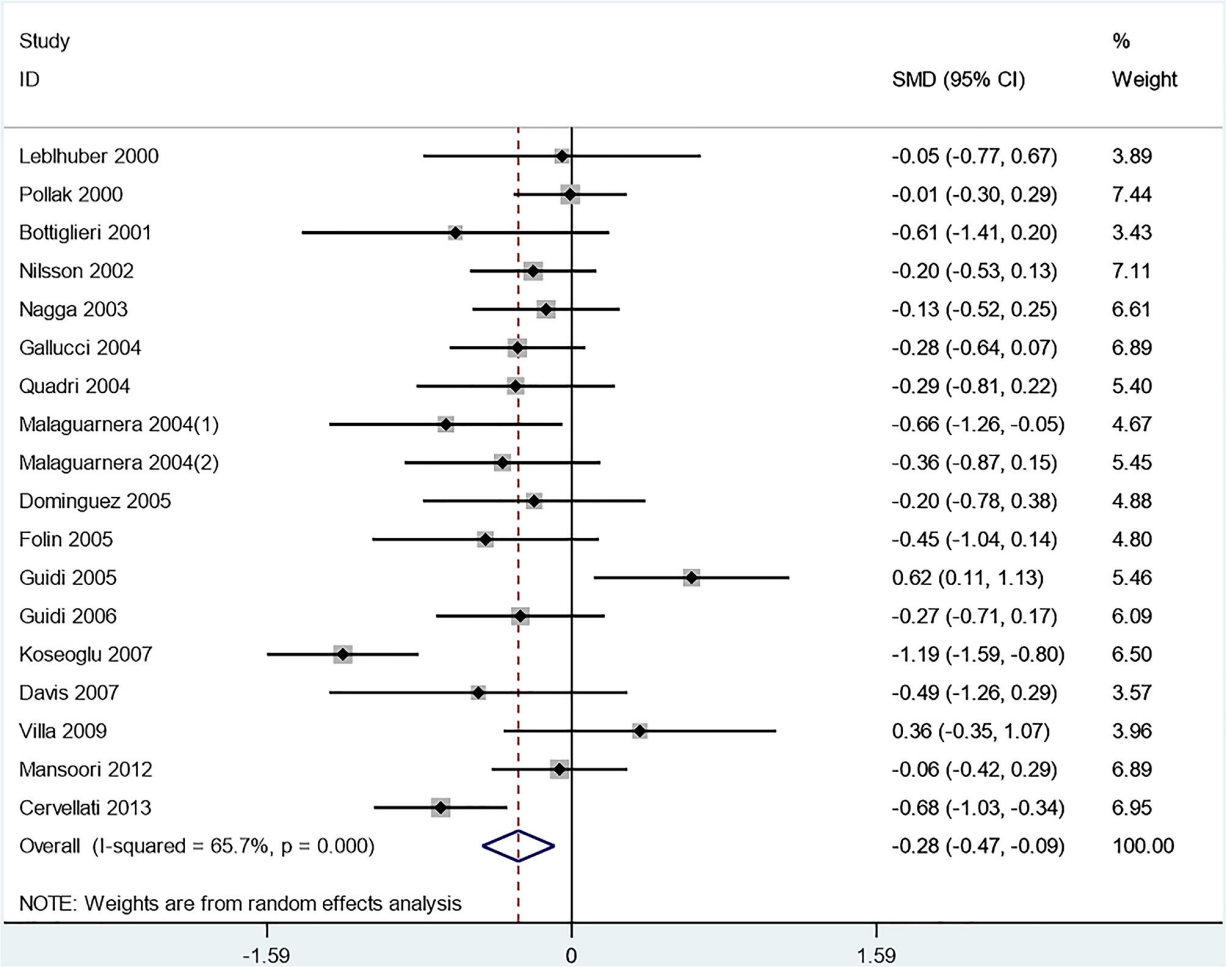
Forest plot of standard mean difference (SMD) and 95% confidence interval (95%CI) in AD and VaD for plasma homocysteine.

A significant reduction in the plasma Hcy level was observed in AD patients compared to VaD patients (SMD = −0.278, 95% CI [−0.466, −0.09], *p* = 0.000) (Figure 4). Additionally, there were no significant differences in folate levels between AD and VaD patients (SMD = 0.032, 95% CI [−0.132, 0.196], *p* = 0.703).

#### Hcy Level and Risk of Dementia

**Figure 5:**
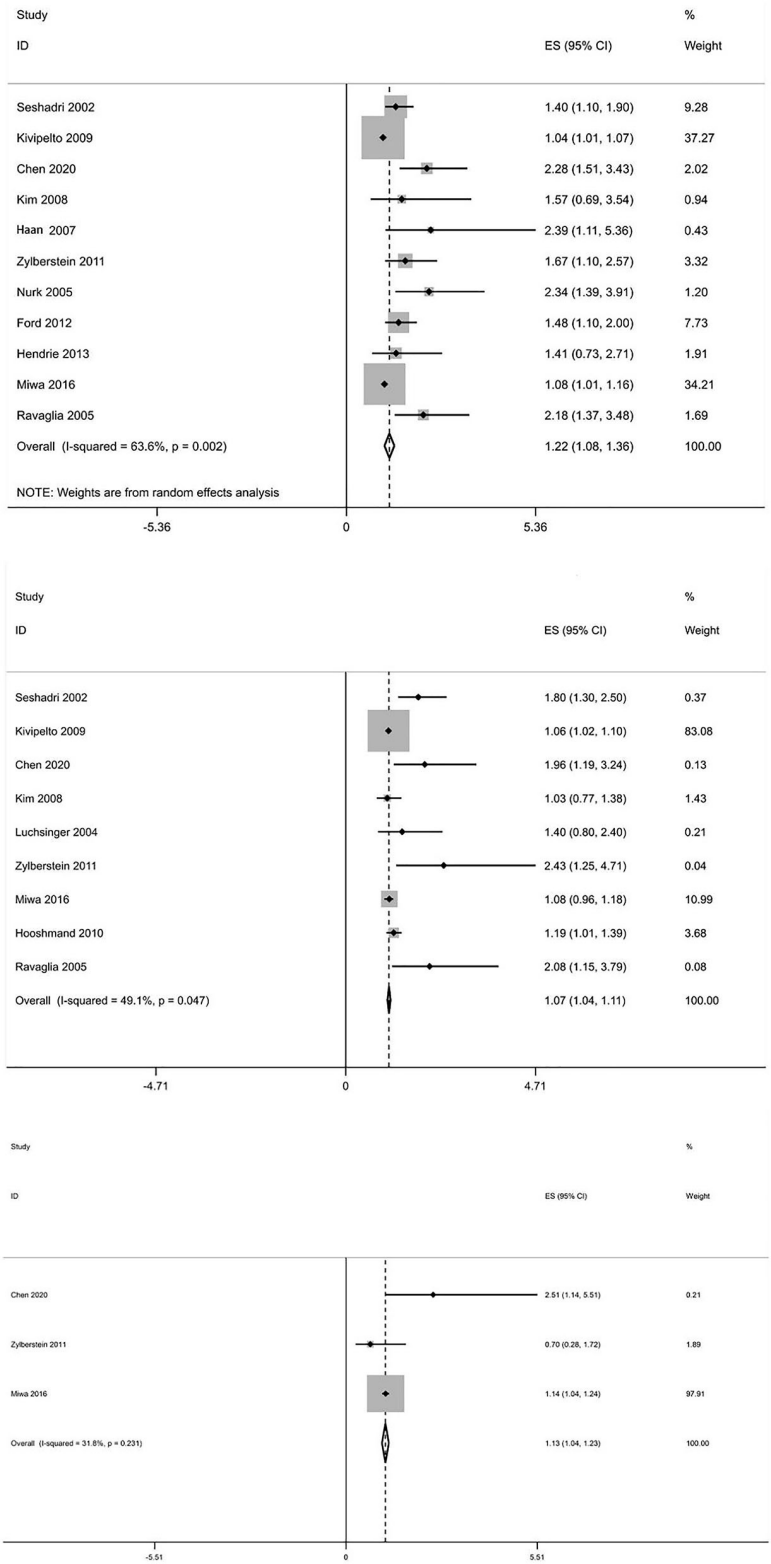
Forest plot of relative risk (RR) and 95% confidence interval (95%CI) in dementia (all-caused dementia, AD, and VaD) and control group for homocysteine.

**Figure 6:**
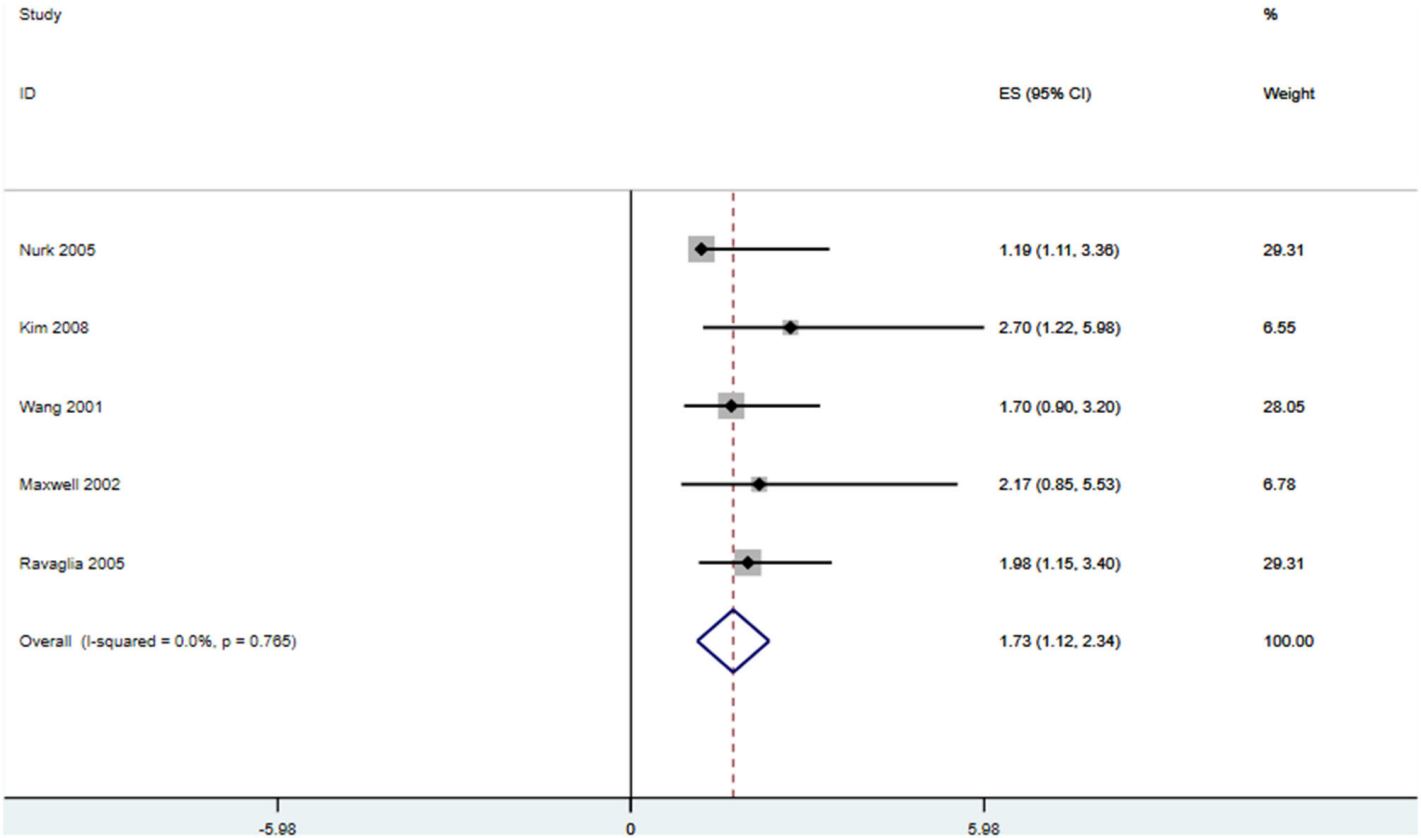
Forest plot of relative risk (RR) and 95% confidence interval (95%CI) in AD and control group for folic acid.

In total, 13 prospective studies on Hcy levels and risk of dementia were included in our study. A summary of these studies is shown in Supplementary Table 1. The pooled overall RR was 1.22, 95% CI (1.08, 1.36), which suggests that an elevated Hcy level may be correlated with a significantly increased risk of all-cause dementia. High serum Hcy levels were also associated with an increased risk of AD and VaD occurrence (RR 1.07, 95% CI [1.04, 1.11]; RR 1.13, 95% CI [1.04, 1.23]) (Figure 5). Five studies were included in our meta-analysis to explore the association between folic acid level and the risk of AD. We found that a low folic acid level was associated with an increased risk of AD (RR 1.731, 95% CI [1.122, 2.34]) (Figure 6).

**Figure 7:**
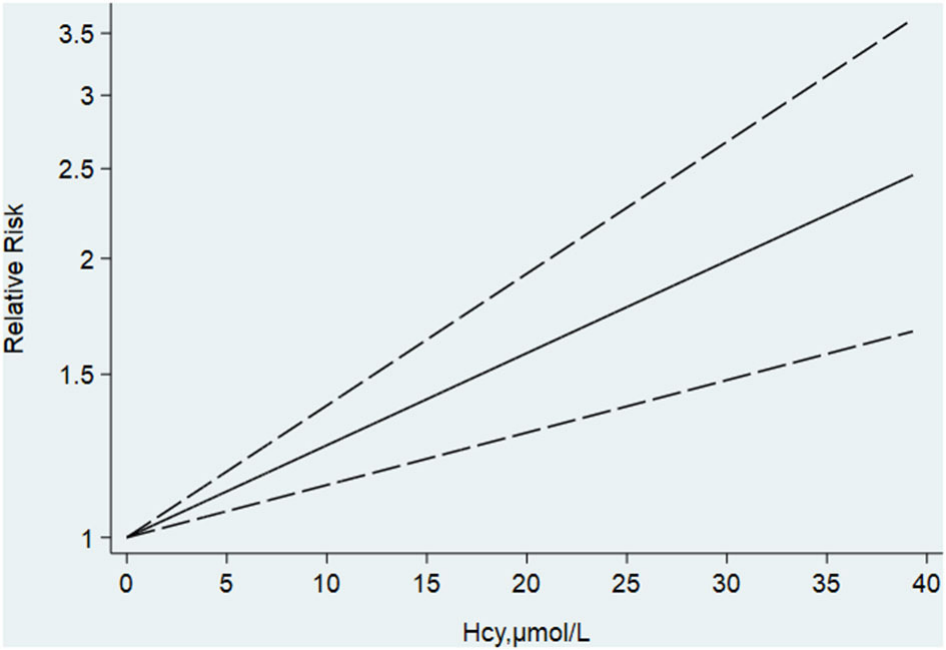
Dose-response analysis of homocysteine and risk of AD. The solid line represents the best fitting cubic spline model.

Begg's and Egger's tests indicated no publication bias. For further linear dose–response analysis, eight studies about Hcy with more than two categories were included, with a total of 15,134 participants and 1,627 all-cause dementia cases. There was no significance for non-linear dose-response analysis (p-non-linear = 0.801). The summary RR of dementia was 1.09 (95% CI [1.057–1.131]) per 5 μmol/L of increased Hcy (Figure 7). In other words, it was estimated that every 5 μmol/L increase in the plasma Hcy level was associated with a 9% increase in the risk of dementia. The summary RR of AD per 5 μmol/L increment in Hcy was 1.12 (95% CI [1.067–1.178]). However, the summary RR was 1.046 (95% CI [0.951–1.14]) for every 5-unit increase with no significance for VaD. In addition, the results of the sensitivity analysis were not substantially different. This indicates that our results are reliable.

## Discussion

Our meta-analysis included 81 articles, and 15 of these were prospective cohort studies. Drawing on this, we systematically analyzed the association between the Hcy and folate levels and the risk of dementia. The results of the meta-analysis not only confirmed that dementia patients had higher Hcy and lower folic acid levels than the controls but also suggested that subjects with high Hcy levels and low folic acid levels might be susceptible to AD. We found a linear trend that for every 5 μmol/L increase in Hcy levels, the risk of dementia and AD increased by 9 and 12%, respectively. To our knowledge, the present meta-analysis is the largest and most comprehensive evaluation of the correlation between Hcy and folate levels and the risk of dementia.

A recent meta-analysis of retrospective studies by Shen et al. demonstrated that AD was significantly associated with high Hcy levels and low serum folate levels. They also suggested that high Hcy and low folate levels may be associated with an increased risk of occurrence of AD (Shen and Ji, 2015). Another meta-analysis by Zhang et al. indicated that all the subtypes of the trial of org 10,172 in acute stroke treatment (TOAST) of ischemic stroke in Chinese patients showed significantly higher Hcy levels compared to the controls (Zhang et al., 1998). Our meta-analysis confirmed that higher levels of Hcy and lower levels of folic acid were observed in patients with dementia than in non-demented controls, which was consistent with the previous meta-analysis results. Further, we found that plasma Hcy levels were higher in VaD patients than in AD patients. In our meta-analysis, we included 26 studies (Fekkes et al., 1998; Pollak et al., 2000; Wang et al., 2001; Maxwell et al., 2002; Malaguarnera et al., 2004; Irizarry et al., 2005; Zhang et al., 2005; Guidi et al., 2006; Davis et al., 2007; Galimberti et al., 2008; Kim et al., 2008a; Bi et al., 2009; Hooshmand et al., 2010; Tu et al., 2010; Ferlazzo et al., 2011; Mansoori et al., 2012, 2014; Piazza et al., 2012; Yesil et al., 2012; Elhawary et al., 2013; Choe et al., 2014; Doody et al., 2015; Madsen et al., 2015; Ma et al., 2017; Soni et al., 2018; Meng et al., 2019; Lanyau-Domínguez et al., 2020; Sutovsky et al., 2020) (21 for Hcy and 13 for folate) that were not included in previous studies. Subgroup analysis by ethnicity revealed that dementia patients in Caucasian and Asian cohorts both had high Hcy levels and low folic acid levels. It was also determined that ethnicity exhibited no significant effect on this association. Further subgroup analysis by average age showed that plasma Hcy levels were significantly higher in dementia patients of every age group than in healthy controls. However, no significant difference was observed in folate levels between dementia patients with an average age of 60–70 years and healthy controls. Recent studies (Hainsworth et al., 2016) showed that older patients need vitamin B supplementation to reduce cognitive impairment, which is in accordance with our results. It seems that folate and vitamin B exhibit a significant role in the dementia of elder people. A previous study indicated that there is a gender difference in the relationship between Hcy levels and the risk of AD. However, our subgroup analysis concluded that both women and men with dementia had higher Hcy levels and lower folate levels than the controls, and the gender exerted no effect on this relationship. Sensitivity analyses suggested that the primary pooled effect size of our analysis remained stable after the exclusion of any single study.

Individual studies on the association between plasma Hcy level and the risk of dementia occurrence did not reach a consensus (Luchsinger et al., 2004; Ford and Almeida, 2012; Miwa et al., 2016). Recently, Zhou et al. performed a meta-analysis of prospective cohort studies and found that an increased level of blood Hcy was associated with the risk of developing AD but not VaD or cognitive impairment (Zhou and Chen, 2019). Our meta-analysis supplemented four studies (Nurk et al., 2005; Haan et al., 2007; Kim et al., 2008b; Chen et al., 2020) and demonstrated a positive association between high levels of Hcy and the risk of dementia, AD, and VaD, which is partly different from the results of the earlier researches. We also identified that higher Hcy levels and lower folate levels were associated with an increased risk of developing dementia and AD. More importantly, a linear dose-dependent relationship between Hcy levels (per 5 μmol/L increase) and the risk of developing AD (12% increase) was found in our study. This finding suggests that every 5 μmol/L increment in plasma Hcy is linearly associated with a 12% increase in AD risk. It has been noted that an increased level of plasma Hcy contributes to the occurrence and development of AD but not VaD.

To date, three trials (FACIT, WAFACS, and VITACOG) have reported the beneficial effects of Hcy-lowering interventions (Hainsworth et al., 2016). The FACIT and WAFACS trials indicated significant effects of B6, B12, and folate on slowing cognitive impairment in patients with high plasma Hcy levels (Douaud et al., 2013). Nieraad et al. suggested that the B-vitamin intervention showed promise to become a preventive therapeutic for AD by the evidence of immediate normalization of Hcy after B-vitamins supplement in the mouse model (Nieraad et al., 2021). Nevertheless, vitamin B supplementation slowed brain atrophy in AD-related regions in subjects with high plasma Hcy levels. It should also be noted that the timing of the Hcy-lowering intervention is critical and that vitamin supplementation exerts a protective effect on AD after 15 months. Hcy as a key mediator in AD development, and Hcy-lowering intervention may be an effective treatment for cognitive impairment.

The following may be the potential Hcy-related mechanisms leading to dementia. First, high Hcy levels can produce superoxide anions, hydrogen peroxide, and hydroxyl anions, which act as promoters of neurodegenerative events and cause endothelial damage and neuronal death (Faraci and Lentz, 2004; Sharma et al., 2015). Moreover, hyperhomocysteinemia is associated with increased hippocampal and cortical atrophy through increasing hippocampal neuron vulnerability to excitotoxic insults and amyloid-peptide toxicity. Hcy may also have a neurotoxic effect on neurons by stimulating the activation of N-methyl-D-aspartate glutamate receptors, resulting in cell death. In addition, hyperhomocysteinemia also affects the synthesis of methionine and S-adenosylmethionine, which could inhibit the methylation reaction, neurotransmitter metabolism, and membrane phospholipids (Lipton et al., 1997; Sachdev et al., 2002). Finally, high Hcy levels can generate β-amyloid peptides through hydrolysis of the amyloid precursor protein. This can lead to the formation of amyloid plaques in neurons (Pacheco-Quinto et al., 2006; Prince et al., 2014). Thus, hyperhomocysteinemia might have versatile roles in cognitive impairment, and complex mechanisms are worthy to be explored. Furthermore, independent of high Hcy levels, folic acid also plays an important role in cognitive function. Folate deficiency causes oxidative stress and generates reactive oxygen species that are responsible for neuronal deterioration and cellular death in areas of the brain involved in AD (Robinson et al., 2018). It has been reported that a folic-acid-deficient diet can promote hippocampal neurodegeneration in amyloid precursor protein mutant transgenic mice (Pacheco-Quinto et al., 2006). Specific mechanisms of Hcy and folate related to the occurrence and development of AD are still not completely lighted, and more exploration is needed to find effective therapy to delay the progression of cognitive decline and dementia.

The strengths of the current study include comprehensive analyses. First, our investigation included prospective studies and retrospective case-control studies providing a comprehensive perspective on the association between Hcy and folate levels and dementia. Second, our current meta-analysis examined the relationship between blood Hcy levels and the risk of cognitive disorders including AD and VaD. Third, we performed subgroup, sensitivity, and dose-response analyses, which provided reliable and precise estimates. Lastly, multiple studies including several cases and participants were included in our study. However, some limitations of our meta-analysis should be considered. First, the heterogeneity among studies was significant owing to differences in geographical location, analytical methods, cut-off values, and patient selection. Second, most studies included in our analysis do not belong to the randomized controlled trial (RCT) category in the strict sense. Randomized placebo-controlled trials of Hcy-lowering treatment are needed to determine the effect of such treatment on the progression of dementia. In addition, although the RR with fully adjusted models was used in our study, different adjustments for confound risk among the different studies could potentially influence the results. Not all the included studies took account of the adjustment for cardiovascular risk factors, MMSE score, vitamin B, duration of follow-up, and other factors. Lastly, only studies with full texts and published papers were included in our meta-analysis; therefore, publication bias may have affected our findings.

In conclusion, our meta-analysis is the first to report that patients with dementia, including AD and VaD, show higher levels of Hcy and lower levels of folic acid compared to non-demented controls. We also revealed a possible influence of elevated plasma Hcy levels and reduced folate levels on the risk of dementia. Moreover, every 5 μmol/L increase in the plasma Hcy level was associated with a 12% increase in the risk of AD. The above findings indicate that Hcy and folic acid may contribute to the incidence of dementia. Our study provides vital evidence for clinicians to rationalize clinical intervention strategies. Moreover, longitudinal studies are needed to further elucidate the relationship between Hcy, folic acid, and dementia.

## Data Availability Statement

The original contributions presented in the study are included in the article/Supplementary Material, further inquiries can be directed to the corresponding author/s.

## Author Contributions

RZ and XL: designed the experiments. RZ, XL, and JZ: performed the experiments. XL, QW, and HC: analyzed the data. RZ and QW: wrote the paper. All authors contributed to the article and approved the submitted version.

## Conflict of Interest

The authors declare that the research was conducted in the absence of any commercial or financial relationships that could be construed as a potential conflict of interest.
