# Supplementary material for: Homocysteine and Folic Acid: Risk Factors for Alzheimer's Disease—An Updated Meta-Analysis
Source: Front Aging Neurosci. 2021 May 26;13:665114. doi: 10.3389/fnagi.2021.665114 (PMC8188894; doi:10.3389/fnagi.2021.665114)
Supplement: Supplementary file 1 [file Table_1.DOCX]

**Supplemental materials**

Table. S1 The quality assessment of Newcastle-Ottawa Scale for included studies.

| Fisrt author and year | Selection | Comparability | Outcome | Summary |
| --- | --- | --- | --- | --- |
| Parnetti 1992 | 3 | 1 | 2 | 6 |
| Joosten 1997 | 3 | 1 | 2 | 6 |
| Clarke 1998 | 4 | 1 | 2 | 7 |
| Fekkes 1998 | 3 | 1 | 2 | 6 |
| Leblhuber 2000 | 3 | 2 | 2 | 7 |
| Pollak 2000 | 4 | 2 | 2 | 8 |
| Bottiglieri 2001 | 3 | 2 | 2 | 7 |
| Postiglione 2001 | 4 | 1 | 2 | 7 |
| Maxwella 2002 | 4 | 2 | 2 | 8 |
| Wang 2001 | 4 | 2 | 1 | 7 |
| Nilsson 2002 | 3 | 2 | 2 | 7 |
| Hogervorst 2002 | 4 | 2 | 2 | 8 |
| Miller 2002 | 3 | 2 | 2 | 7 |
| Seshadri 2002 | 4 | 2 | 3 | 9 |
| Selley 2002 | 3 | 2 | 2 | 7 |
| Selley 2003 | 3 | 2 | 2 | 7 |
| Mizrahi 2003 | 4 | 1 | 2 | 7 |
| Nagga 2003 | 3 | 2 | 2 | 7 |
| Religa 2003 | 4 | 2 | 2 | 8 |
| Gallucci 2004 | 4 | 2 | 2 | 8 |
| Genedani 2004 | 3 | 1 | 2 | 6 |
| Mizrahi 2004 | 4 | 1 | 2 | 7 |
| Quadri 2004 | 4 | 2 | 2 | 8 |
| Anello 2004 | 4 | 2 | 2 | 8 |
| Ravaglia 2004 | 3 | 2 | 2 | 7 |
| Luchsinger 2004 | 4 | 2 | 2 | 8 |
| Malaguarnera 2004(1) | 3 | 2 | 2 | 7 |
| Malaguarnera 2004(2) | 3 | 2 | 2 | 7 |
| Irizarry 2005 | 4 | 2 | 2 | 8 |
| Dominguez 2005 | 3 | 2 | 2 | 7 |
| Folin 2005 | 3 | 2 | 2 | 7 |
| Zhang 2005 | 4 | 1 | 2 | 7 |
| Guidi 2005 | 3 | 2 | 2 | 7 |
| Nurk 2005 | 4 | 2 | 3 | 9 |
| Ravaglia 2005 | 4 | 2 | 2 | 8 |
| Asita De Silva 2005 | 3 | 1 | 2 | 6 |
| Quadri 2005 | 4 | 1 | 2 | 7 |
| Guidi 2006 | 4 | 2 | 2 | 8 |
| Selly 2006 | 3 | 1 | 2 | 6 |
| da Silva 2006 | 3 | 1 | 2 | 6 |
| Hernanaz 2007 | 3 | 2 | 2 | 7 |
| Koseoglu 2007 | 3 | 2 | 2 | 7 |
| Davis 2007 | 3 | 2 | 2 | 7 |
| Kim 2007 | 4 | 1 | 2 | 7 |
| Lovati 2007 | 4 | 1 | 2 | 7 |
| Hann 2007 | 4 | 1 | 2 | 7 |
| Galimberti 2008 | 3 | 1 | 2 | 6 |
| Hagnelius 2008 | 3 | 2 | 2 | 7 |
| Kim 2008 | 4 | 2 | 2 | 8 |
| Villa 2009 | 3 | 2 | 2 | 7 |
| Karimi 2009 | 4 | 1 | 2 | 7 |
| Bi 2009 | 4 | 1 | 2 | 7 |
| Kivipelto 2009 | 3 | 2 | 3 | 8 |
| Linnebank 2010 | 4 | 1 | 2 | 7 |
| Tu 2010 | 4 | 1 | 2 | 7 |
| Morillas-Ruiz 2010 | 3 | 1 | 2 | 6 |
| Hooshmand 2010 | 3 | 2 | 2 | 7 |
| Smach 2011 | 3 | 2 | 2 | 7 |
| Ferlazzo 2011 | 4 | 2 | 2 | 8 |
| Faux 2011 | 4 | 2 | 2 | 8 |
| Zylberstein 2011 | 4 | 2 | 3 | 9 |
| Czapski 2012 | 4 | 2 | 2 | 8 |
| Mansoori 2012 | 4 | 2 | 2 | 8 |
| Yesil 2012 | 4 | 1 | 2 | 7 |
| Piazza 2012 | 4 | 2 | 2 | 8 |
| Ford 2012 | 4 | 1 | 3 | 8 |
| Kim 2013 | 4 | 2 | 2 | 8 |
| Elhawary 2013 | 3 | 1 | 2 | 6 |
| Cervellati 2013 | 4 | 2 | 2 | 8 |
| Hendrie 2013 | 4 | 1 | 3 | 8 |
| Choe 2014 | 3 | 2 | 2 | 7 |
| Madsen 2014 | 4 | 2 | 2 | 8 |
| Mansoori 2014 | 4 | 2 | 2 | 8 |
| Doody 2015 | 4 | 1 | 2 | 7 |
| Miwa 2016 | 4 | 2 | 3 | 9 |
| Ma 2017 | 4 | 2 | 2 | 8 |
| Soni 2018 | 3 | 1 | 2 | 6 |
| Meng 2019 | 4 | 1 | 2 | 7 |
| Yeneisy 2020 | 3 | 2 | 2 | 7 |
| Sutovsky 2020 | 4 | 1 | 2 | 7 |
| Chen 2020 | 4 | 2 | 3 | 9 |

**Table S2**

Risk relative for dementia in studies included in prospective cohort studies on homocysteine concentration (μmol/L).

| Study | country | Sample | Case | Follw  -up | Sample  (group） | Case  (group) | Hcy exposure | Hcy mean | adrr | Disease type | Adjustment for other risk factors |
| --- | --- | --- | --- | --- | --- | --- | --- | --- | --- | --- | --- |
| Seshadri 2002 | USA | 680 | 60 | 8 | / | / | >14 | / | 1.4 (1.1–1.9) | All-cause-dementia | Age, gender, education, VitB, BP, smoking, stroke, Apo E and others |
|  |  | 680 | 44 | 8 | / | / | >14 | / | 1.8 (1.3–2.5) | AD |  |
| Kivipelto 2009 | Sweden | 213 | 88 | 6.7 | / | / | >14 | / | 1.04(1.01–1.07) | All-cause-dementia | Age, sex, education, BMI, albumin, haemoglobin, creatinine, ApoE, MMSE score, holo-TC, VitB12 and folate. |
|  |  | 213 | 88 | 6.7 | 53 | 19 | 5-8.7 | 6.85 | 1 | All-cause-dementia |  |
|  |  |  |  |  | 53 | 14 | 8.8-12.5 | 10.65 | 0.83 (0.39-1.77) |  |  |
|  |  |  |  |  | 53 | 15 | 12.6-16.3 | 14.45 | 1.12 (0.51-2.42) |  |  |
|  |  |  |  |  | 54 | 35 | 16.4-20 | 18.2 | 1.79 (0.86-3.74) |  |  |
|  |  | 213 | 61 | 6.7 | / | / | >14 | / | 1.06(1.02-1.1) | AD |  |
|  |  | 213 | 61 | 6.7 | 53 | 13 | 5-8.7 | 6.85 | 1 | AD |  |
|  |  |  |  |  | 53 | 8 | 8.8-12.5 | 10.65 | 0.71 (0.26-1.91) |  |  |
|  |  |  |  |  | 53 | 12 | 12.6-16.3 | 14.45 | 1.45 (0.57-3.69) |  |  |
|  |  |  |  |  | 54 | 28 | 16.4-20 | 18.2 | 2.57 (1.06-6.24) |  |  |
| Chen 2020 | Janpanese | 1588 | 372 | 1.2 | 329 | 54 | ≤6.4 | 3.2 | 1 | All-cause-dementia | Age, sex, education, HBP, DM, serum TC, BMI, estimated GFR, history of stroke, current smoking, current drinking, regular exercise, serum albumin, serum folate and VitB12 levels |
|  |  |  |  |  | 298 | 55 | 6.5–7.6 | 7.05 | 1.13 (0.77-1.66) |  |  |
|  |  |  |  |  | 332 | 68 | 7.7–9.0 | 8,35 | 1.25 (0.85-1.82) |  |  |
|  |  |  |  |  | 312 | 80 | 9.1–11.4 | 10.25 | 1.38 (0.93-2.03) |  |  |
|  |  |  |  |  | 317 | 115 | ≥11.5 |  | 2.28 (1.51-3.43) |  |  |
|  |  | 1588 | 247 | 10.2 | 329 | 40 | ≤6.4 | 3.2 | 1 | AD |  |
|  |  |  |  |  | 298 | 39 | 6.5–7.6 | 7.05 | 1.13 (0.72-1.78) |  |  |
|  |  |  |  |  | 332 | 46 | 7.7–9.0 | 8,35 | 1.12 (0.71-1.76) |  |  |
|  |  |  |  |  | 312 | 53 | 9.1–11.4 | 10.25 | 1.28 (0.80-2.04) |  |  |
|  |  |  |  |  | 317 | 69 | ≥11.5 |  | 1.96 (1.19-3.24) |  |  |
|  |  | 1588 | 98 | 10.2 | 329 | 14 | ≤6.4 | 3.2 | 1 | VAD |  |
|  |  |  |  |  | 298 | 9 | 6.5–7.6 | 7.05 | 0.64 (0.27-1.52) |  |  |
|  |  |  |  |  | 332 | 17 | 7.7–9.0 | 8,35 | 1.37 (0.64-2.94) |  |  |
|  |  |  |  |  | 312 | 19 | 9.1–11.4 | 10.25 | 1.21 (0.56-2.63) |  |  |
|  |  |  |  |  | 317 | 39 | ≥11.5 |  | 2.51 (1.14-5.51) |  |  |
| Kim 2008 | Korea | 518 | 45 | 2.4 | / | / | / | 6.4 | 1 | All-cause-dementia | Age, sex, education, VitB, smoking and others |
|  |  |  |  |  |  |  |  | 9.1 | 1.08 (0.83–1.41) |  |  |
|  |  |  |  |  |  |  |  | 11.3 | 1.17 (0.69–1.99) |  |  |
|  |  |  |  |  |  |  |  | 14.1 | 1.26 (0.57–2.80) |  |  |
|  |  |  |  |  |  |  |  | 20.4 | 1.36 (0.47–3.95) |  |  |
|  |  | 518 | 45 | 2.4 | / | / | >13.9 |  | 1.57（0.69-3.54) | All-cause-dementia | Age, sex, education, VitB, smoking and others |
|  |  | 518 | 45 | 2.4 | / | / | >13.9 |  | 1.03(0.77-1.38) | AD |  |
| Hann 2007 | USA | 1779 | 62 | 4.5 | / | / | / | 4.7 | 1 | All-cause-dementia | Age, sex, education, VitB, stroke |
|  |  |  |  |  |  |  |  | 7.7 | 1.45 (0.96–2.32) |  |  |
|  |  |  |  |  |  |  |  | 11 | 2.02 (0.92–4.81) |  |  |
|  |  |  |  |  |  |  |  | 19.3 | 3.94 (0.85-21.64) |  |  |
|  |  | 1779 | 62 | 4.5 | / | / | > 13 | / | 2.39（1.11-5.36） | All-cause-dementia | Age, sex, education, VitB, stroke |
| Luchsinger 2004 | USA | 679 | 109 | 4.7 | / | / | / | / | 1.4(0.8-2.4) | AD |  |
|  |  | 679 | 109 | 4.7 | 177 | 26 | / | 10.75 | 1 | AD | Age, sex, education, stroke, Apo E |
|  |  |  |  |  | 184 | 29 | / | 14.09 | 1.10 (0.7-2.0) |  |  |
|  |  |  |  |  | 164 | 24 | / | 17.52 | 1 (0.6-1.8) |  |  |
|  |  |  |  |  | 154 | 30 | / | 27.44 | 1.3 (0.8-2.3) |  |  |
| Ravaglia 2005 | Italy | 816 | 112 | 4 | / | / | > 15 | / | 2.18(1.37-3.48) | All-cause-dementia |  |
|  |  | 816 | 112 | 4 | / | / | > 15 | / | 2.08(1.15-3.79) | AD |  |
|  |  | 816 | 112 | 4 | 211 | 13 | < 10.1 | 6.73 | 1 | All-cause-dementia | Age, gender, education, B vitamins, blood pressure, smoking, stroke, Apo E, others |
|  |  |  |  |  | 204 | 23 | 10.1-12.5 | 11.3 | 1.7 (0.6-3.5) |  |  |
|  |  |  |  |  | 184 | 21 | 12.6-15.0 | 13.8 | 2.1 (0.9-4.1) |  |  |
|  |  |  |  |  | 217 | 55 | > 15.0 | 22.5 | 3.5 (1.7-7.5) |  |  |
|  |  | 816 | 112 | 4 | 211 | 8 | < 10.1 | 6.73 | 1 | AD |  |
|  |  |  |  |  | 204 | 17 | 10.1-12.5 | 11.3 | 2.5 (0.93-6.0) |  |  |
|  |  |  |  |  | 184 | 14 | 12.6-15.0 | 13.8 | 2.5 (0.93-6.1) |  |  |
|  |  |  |  |  | 217 | 31 | > 15.0 | 22.5 | 4.2 (1.7-11.0) |  |  |
| Zylberstein 2011 | Sweden | 1368 | 151 | 30 | 254 | 39 | 3.1-9.8 | 6.45 | 1 | All-cause-dementia | Age, education, B vitamins, blood pressure and others |
|  |  |  |  |  | 564 | 49 | 9.8-12.6 | 11.2 | 1.3 (0.84-2.0) |  |  |
|  |  |  |  |  | 441 | 63 | 12.6-78.9 | 45.75 | 1.67 (1.10-2.57) |  |  |
|  |  | `1368 | 151 | 30 | 254 | 12 | 3.1-9.8 | 6.45 | 11 | AD |  |
|  |  |  |  |  | 564 | 40 | 9.8-12.6 | 11.2 | 1.54 (0.78-3.05) |  |  |
|  |  |  |  |  | 441 | 46 | 12.6-78.9 | 45.75 | 2.43 (1.25-4.71) |  |  |
|  |  | `1368 | 151 | 30 | 254 | 9 | 3.1-9.8 | 6.45 | 11 | VAD |  |
|  |  |  |  |  | 564 | 15 | 9.8-12.6 | 11.2 | 0.76 (0.35-1.64) |  |  |
|  |  |  |  |  | 441 | 13 | 12.6-78.9 | 45.75 | 0.70 (0.28-1.72) |  |  |
| Nurk 2005 | Norway | 2189 | 235 | 6 | 452 | 33 | / | 8.3 | 1 | Memory Deficit (Kendrick Object Learning Test score 25) | Adjusted for sex, Apo E, ε4 alleles, education, history of cardiovascular diseases and HBP. In addition, the results at follow-up were adjusted for depression score. |
|  |  |  |  |  | 441 | 37 | / | 10 | 1.05 (0.58–1.89) |  |  |
|  |  |  |  |  | 472 | 50 | / | 11.5 | 1.7 (1.01–2.88) |  |  |
|  |  |  |  |  | 387 | 51 | / | 13.3 | 1.66 (0.95–2.91) |  |  |
|  |  |  |  |  | 432 | 63 | / | 16.5 | 2.34 (1.39-3.91) |  |  |
| Ford 2012 | Australia | 2959 | 228 | 5.8 | / | / | / | / | 1.48(1.1-2) | All-cause-dementia |  |
|  |  | 2959 | 228 | 5.8 | 1033 | 43 | ≤10.3 | 6.87 | 1 | All-cause-dementia |  |
|  |  |  |  |  | 995 | 48 | / | 12.6 | 0.88 (0.57-1.36) |  |  |
|  |  |  |  |  | 988 | 65 | / | 13.9 | 1.06 (0.70-1.61) |  |  |
|  |  |  |  |  | 981 | 74 | > 15.1 | 22.65 | 1.2 (0.8-1.79) |  |  |
| Hendrie 2013 | USA | 819 | 101 | 5.1 | 283 | 19 | 4.44-10.71 | 7.58 | 1 | All-cause-dementia | Age, history of ischaemic heart disease and of stroke |
|  |  |  |  |  | 284 | 22 | 10.72-16.99 | 13.86 | 1.16 (0.58-2.28) |  |  |
|  |  |  |  |  | 284 | 32 | 17.0-23.27 | 20.14 | 1.78 (0.94-3.38) |  |  |
|  |  |  |  |  | 284 | 28 | 23.28-29.52 | 26.4 | 1.41 (0.73-2.71) |  |  |
| Miwa 2016 | Japan | 643 | 47 | 7.3 | / | / | / | / | 1.08(1.01-1.16) | All-cause-dementia |  |
|  |  | 643 | 47 | 7.3 | 214 | 11 | ≤8.2 | 5.47 | 1 | All-cause-dementia | Age, sex, education, APOE-Ɛ4, BMI, MMSE, HBP, previous cerebrovascular diseases |
|  |  |  |  |  | 214 | 9 | 8.3-10.7 | 9.5 | 0.78 (0.27-1.94) |  |  |
|  |  |  |  |  | 215 | 27 | ≥10.8 | 16.2 | 2.5 (1.01-6.63) |  |  |
|  |  | 643 | 24 | 7.3 | / | / | / | / | 1.08(0.96-1.18) | AD |  |
|  |  | 643 | 24 | 7.3 | 214 | 1 | ≤8.2 | 5.47 | 1 | AD |  |
|  |  |  |  |  | 214 | 1 | 8.3-10.7 | 9.5 | 0.71 (0.09-4.49) |  |  |
|  |  |  |  |  | 215 | 22 | ≥10.8 | 16.2 | 3.31 (0.82-17.13) |  |  |
|  |  | 643 | 21 | 7.3 | / | / | / | / | 1.14(1.04-1.24) | VAD |  |
|  |  | 643 | 21 | 7.3 | 214 | 3 | ≤8.2 | 5.47 | 1 | VAD |  |
|  |  |  |  |  | 214 | 4 | 8.3-10.7 | 9.5 | 1.29 (0.15-6.48) |  |  |
|  |  |  |  |  | 215 | 14 | ≥10.8 | 16.2 | 6.29 (1.10-18.42) |  |  |
| Hooshmand et al., 2010 | Sweden | 271 | 17 | 7 | / | / | > 12.3 | / | 1.19 (1.01-1.39) | AD | Age, sex, education, duration of follow up, APOE-Ɛ4, BMI, MMSE, SBP/D |

Vit: vitamin; BP: blood pressure; BMI: body mass index; HBP: hypertension; GFR: glomerular filtration rate; DM: diabetes mellitus; TC: total cholesterol; MMSE: mini-mental state examination; ApoE: apolipoprotein E

**Table S3**

The baseline characteristics of included studies for VaD (homocysteine).

| First author | Country | Method | AD | | | Controls | | |
| --- | --- | --- | --- | --- | --- | --- | --- | --- |
|  |  |  | N(male) | age | Hcy | N(male) | age | Hcy |
| Leblhuber_VAD 2000 | Caucasian | FPIA | 12(5) | 74.3±7.6 | 18.5±7.8 | 19(8) | 70.2±8.8 | 13.8±4.2 |
| Pollak_VAD 2000 | Caucasian | amino acid analysis | 85(33) | 84±6.3 | 12±3.0 | 82(21) | 82±8.2 | 14±7.1 |
| Bottiglieri_VAD 2001 | Caucasian | HPLC | 7(4) | 72.9±6.2 | 19.6±16.8 | 14(9) | 40.6±14.6 | 7.1±2.9 |
| Nilsson_VAD 2002 | Caucasian | HPLC | 57 | 80.5±6.8 | 20.2±6.8 | 36 | 79.9±3.7 | 15.5±3.7 |
| Nagga_VAD 2003 | Caucasian | FPIA | 59(22) | 78.4±5.4 | 17.3±5.6 | 101(52) | 69.0±5.8 | 12.9±4.2 |
| Gallucci_VAD 2004 | Caucasian | HPLC | 40(21) | 80.3±6.0 | 24.4 ±10.6 | 42(16) | 76.8±9.7 | 15.5±5.2 |
| Quadri_VAD 2004 | Caucasian | FPIA | 18(7) | 80.5±5.7 | 18.9±7.9 | 55(21) | 75.6±8.5 | 14.6±6.1 |
| Malaguarnera _VAD 2004(1) | Caucasian | HPLC | 22(13) | 75.8±6.47 | 26.0±6.58 | 24(12) | 73.7±4.20 | 10.7±3.00 |
| Malaguarnera _VAD 2004(2) | Caucasian | HPLC | 30(17) | 74.7±7.83 | 25.5±6.33 | 30(13) | 73.6±4.14 | 10.8±2.92 |
| Dominguez_VAD 2005 | Caucasian | FPIA | 19(11) | 74.32±5.14 | 19.29±5.60 | 19(12) | 73.89±8.87 | 11.11±1.88 |
| Folin _VAD 2005 | Caucasian | HPLC | 13 | 82.46±4.75 | 24.54±8.12 | 24 | 71.24±9.69 | 15.79±5.55 |
| Guidi_VAD 2005 | Caucasian | FPIA | 18 | ≤65 | 14.3±5.09 | 23 | 71 | 13.0±3.36 |
| Guidi _VAD 2006 | Caucasian | FPIA | 28(15) | 78 (63–90) | 21.05 ± 7.57 | 44(10) | 73 (54–93) | 12.89 ± 4.11 |
| Koseoglu_VAD 2007 | Caucasian | HPLC | 67(42) | 80 ± 4.79 | 18.6 ± 4.15 | 40(17) | 76.13±3.88 | 10.3±1.28 |
| Davis_VAD 2007 | Caucasian | FPIA | 10 | / | 11.66 ± 3.38 | 46 | 69.5±6.17 | 9.85 ± 2.17 |
| Villa_VAD 2009 | Caucasian | HPLC/fluorescence detection | 15(8) | 71.3±7.16 | 13.1±1.99 | 15(7) | 74.7±6.73 | 9.1±2.82 |
| Mansoori_VAD 2012 | Asian | competitive IA | 50(37) | 65.4±9.1 | 15.81±5.55 | 120(75) | 63.8±8.2 | 13.55±5.4 |
| Cervellati_VAD 2013 | Caucasian | ROCHE COBAS INTEGRA 800 chemistry analyzer | 54(24) | 79.3 ± 0.5 | 18.1 ± 2.2 | 48(15) | 77.8 ± 0.7 | 14.6 ± 3.4 |

**Table S4**

The baseline characteristics of included studies for VaD (folic acid).

| First author | Ethnicity | Source | Method | VAD | | | Controls | | |
| --- | --- | --- | --- | --- | --- | --- | --- | --- | --- |
|  |  |  |  | n(male) | age | Folic acid(nM/L) | n(male) | age | Folic acid(nM/L) |
| Parnetti_VAD 1992 | Caucasian | plasma | RIA | 28 | 73±6.88 | 8.85±4.80 | 26 | 72.1 ± 1.4 | 14.07± 5.79 |
| Leblhuber_VAD 2000 | Caucasian | serum | RIA | 12(5) | 74.3±7.6 | 9.31±4.09 | 19(8) | 70.2±8.8 | 14.30±9.31 |
| Bottiglieri_VAD 2001 | Caucasian | plasma | RIA | 7(4) | 72.9±6.2 | 10.7±15.5 | 14(9) | 40.6±14.6 | 12.1±10.0 |
| Quadri_VAD 2004 | Caucasian | serum | RIA | 18(7) | 80.5±5.7 | 14.0±5.9 | 55(21) | 75.6 ± 8.5 | 16.9 ± 5.8 |
| Malaguarnera_VAD 2004(1) | Caucasian | serum | RA | 22(13) | 75.8±6.47 | 10.8±2.81 | 24(12) | 73.7±4.20 | 13.9±3.03 |
| Malaguarnera _VAD 2004(2) | Caucasian | serum | RA | 30(17) | 74.7±7.83 | 11.9±3.28 | 30(13) | 73.6 ± 4.1 | 13.6 ± 3.18 |
| Gallucci_VAD 2004 | Caucasian | serum | chemiluminescence | 40(21) | 80.3±6.0 | 9.53±4.77 | 42(12) | 76.8±9.7 | 14.07±11.12 |
| Dominguez_VAD 2005 | Caucasian | serum | ionic capture assay | 19(11) | 74.32±5.14 | 16.46±7.60 | 19(12) | 73.89±8.87 | 29.62± 8.99 |
| Koseoglu_VAD 2007 | Caucasian | serum | competitive CLIA | 67(42) | 80 ± 4.79 | 19.98±3.61 | 40(17) | 76.13±3.88 | 28.15±3.41 |
| Villa_VAD 2009 | Caucasian | plasma | chemiluminescent | 15(8) | 71.3±7.16 | 18.84±7.65 | 15(7) | 74.7 ± 6.73 | 19.07 ± 4.09 |
| Mansoori_VAD 2014 | Asian | serum | competitive IA | 50(37) | 65.4±9.0 | 17.25±5.90 | 120(75) | 63.8±8.2 | 19.98±8.17 |

Fig. S1. Galbraith graph of heterogeneity analysis in the meta-analysis (left: homocysteine; right: folic acid).

Fig. S2. Begg’s funnel plot of publication bias in the meta-analysis (left: homocysteine; right: folic acid).

Fig. S3. Sensitivity analysis plot of our meta-analysis for folic acid.

Fig.S4. Sensitivity analysis plot of our meta-analysis for homocysteine.

Fig. S5. Forest plot of subgroup analysis on dementia type (AD and VaD) of standard mean difference (SMD) and 95% confidence interval (95%CI) in dementia and control group for homocysteine.

Fig. S6. Forest plot of subgroup analysis on ethnicity (Caucasian or Asia) of standard mean difference (SMD) and 95% confidence interval (95%CI) in dementia and control group for homocysteine.

Fig. S7. Forest plot of subgroup analysis on average age (60 ≤ age < 70, 70 ≤age < 80 or age ≥ 80) of standard mean difference (SMD) and 95% confidence interval (95%CI) in dementia and control group for homocysteine.


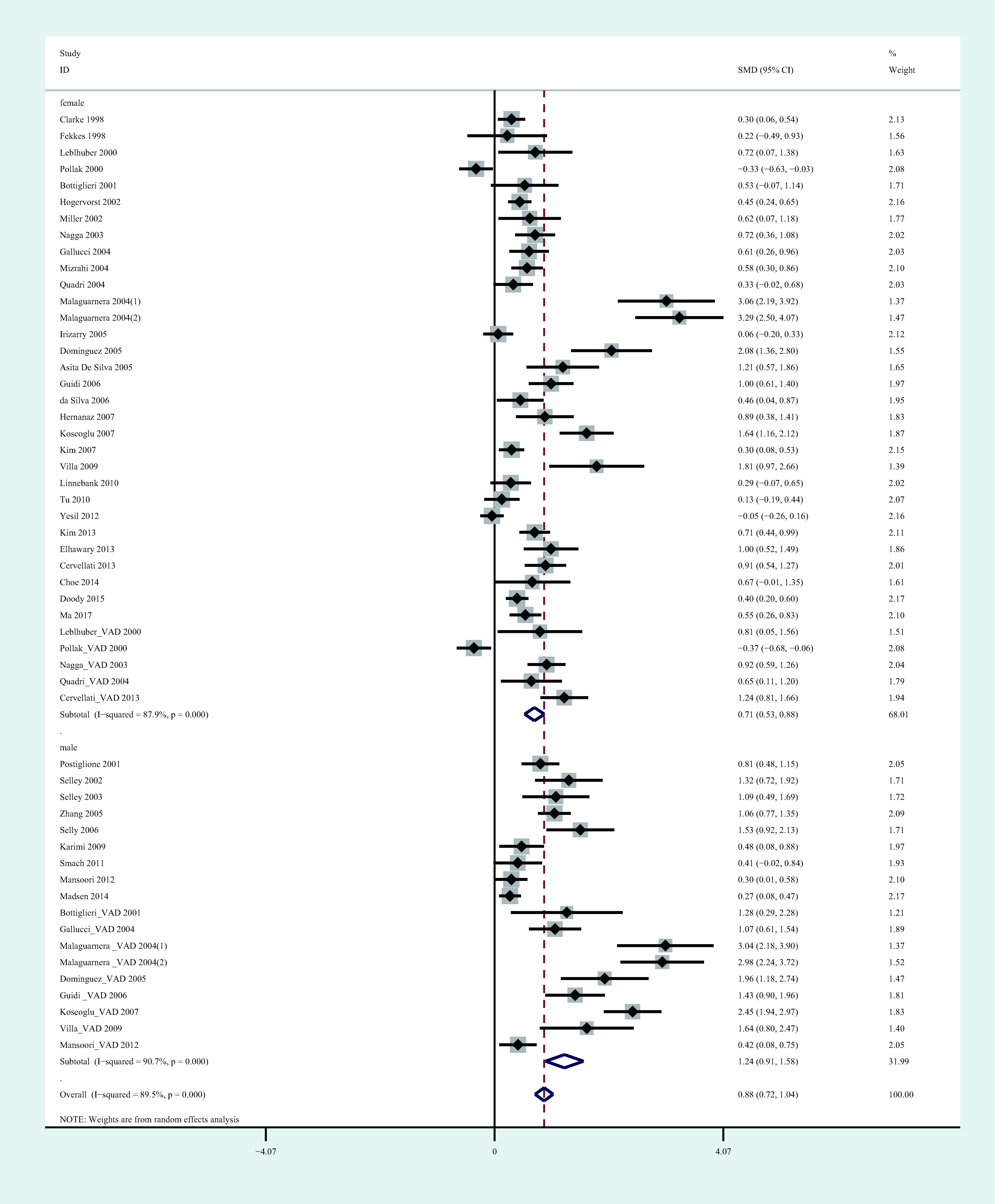


Fig. S8. Forest plot of subgroup analysis on gender (male or female) of standard mean difference (SMD) and 95% confidence interval (95%CI) in dementia and control group for homocysteine.

Fig. S9. Forest plot of subgroup analysis on dementia type (AD and VaD) of standard mean difference (SMD) and 95% confidence interval (95%CI) in dementia and control group for folic acid.

Fig. S10. Forest plot of subgroup analysis on ethnicity (Caucasian or Asia) of standard mean difference (SMD) and 95% confidence interval (95%CI) in dementia and control group for folic acid.

Fig. S11. Forest plot of subgroup analysis on average age (60 ≤ age < 70, 70 ≤age < 80 or age ≥ 80) of standard mean difference (SMD) and 95% confidence interval (95%CI) in dementia and control group for folic acid.


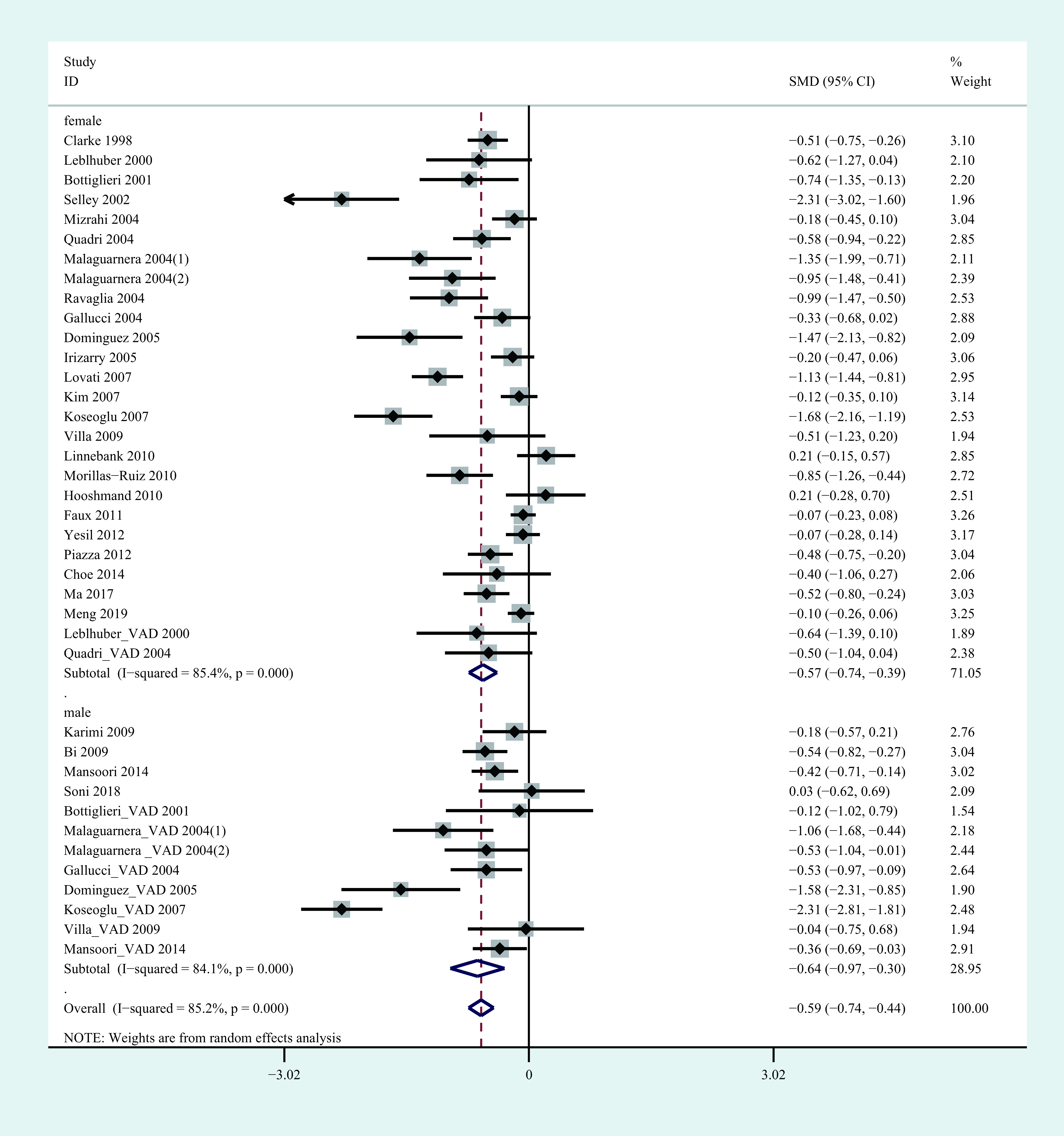


Fig. S12. Forest plot of subgroup analysis on gender (male or female) of standard mean difference (SMD) and 95% confidence interval (95%CI) in dementia and control group for folic acid.
